# A tetrathiafulvalene-containing covalent organic nanobelt: preparation, crystal structure and application for sodium-ion batteries†

**DOI:** 10.1039/d4sc06300g

**Published:** 2024-11-14

**Authors:** Xin Wang, Yuchan Zhang, Lei Zhang, Qianfeng Gu, Qi Liu, Yang Ren, Chun Sin Lee, Qichun Zhang

**Affiliations:** a Department of Materials Science and Engineering, City University of Hong Kong Hong Kong SAR 999077 P. R. China qiczhang@cityu.edu.hk; b Department of Physics, City University of Hong Kong Hong Kong SAR 999077 P. R. China; c Department of Chemistry, Center of Super-Diamond and Advanced Films (COSDAF), Hong Kong Institute of Clean Energy, City University of Hong Kong Hong Kong SAR 999077 P. R. China

## Abstract

Developing single crystals of covalent organic polymers (COPs) is highly attractive as they can afford precise structural information for studying internal interactions. Employing dative boron–nitrogen (B–N) bonds to construct single-crystalline COPs is feasible since the dynamic linkages can self-correct errors, thus improving crystallization. In this project, we develop a single-crystal COP with a nanobelt structure, namely CityU-26, *via* B–N-driven-assembly between 4,4′,5,5′-tetrakis(4-(pyridin-4-yl)phenyl)-2,2′-bi(1,3-dithiolylidene) and 1,4-bis(benzodioxaborole) benzene. The B–N coordination between these units gives rise to one-dimensional (1D) nanobelts, and hydrogen bonding interactions between the nanobelts lead to the formation of a three-dimensional (3D) supramolecular structure. CityU-26 demonstrates an impressive sodium storage capability of 365 mA h g^−1^ with a current density of 150 mA g^−1^, and the capability could reach 315 mA h g^−1^ at 750 mA g^−1^. The outstanding sodium storage behaviors of CityU-26 underscore the functionalization of B–N polymers, providing a promising platform for the development of efficient energy materials.

## Abstract 2

Single crystals of covalent organic polymers (CityU-26) with a nanobelt structure were obtained *via* B–N-driven assembly, demonstrating an impressive sodium storage capability of 365 mA h g^−1^ at 150 mA g^−1^.

## Graphical abstract

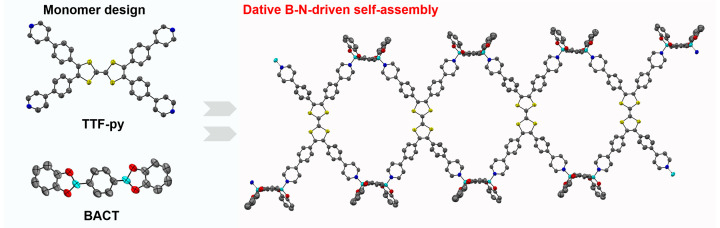

## Introduction

Covalent organic polymers (COPs) have gained significant interest in diverse fields such as automobiles and energy storage due to the advantages of light weight, flexibility, and ease of manufacturing.^1–3^ However, a deep and comprehensive understanding of the structure–property relations is still missing,^4^ as most COPs are crystalline powders or amorphous solids.^5,6^ The structure determination is usually based on their powder X-ray diffraction (PXRD) patterns, but most simulation results cannot provide exact structural information.^7^ Therefore, growing single crystals of COPs for single-crystal XRD (SCXRD) analysis has been the subject of extensive study since they can provide precise information for studying inter- and intra-molecular interactions as well as their stacking.^8–10^ The unambiguous molecular arrangement is helpful in the investigation of structure–performance relationships, promoting the development of novel COPs.^11,12^ To date, several linkages such as azodioxy,^13^ boronic ester,^14^ and amine bonds^15,16^ have been used to prepare single crystals of COPs. The reversible nature of these linkages allows for the self-correction of errors during the crystallization process, facilitating the appearance of single crystals.^17^

The critical factor for growing single crystals of COPs is the utilization of reversible bonds. Motivated by previous reports,^18^ we turned our attention to dative boron–nitrogen (B–N) bonds for their covalent nature, reversibility, and tunability,^19–21^ which would encourage the realization of single crystals of COPs. Typically, B–N linkages come from interactions between Lewis pairs.^22^ Since B–N bonds are rather exchangeable in solution but become robust in the solid state,^23^ their reversible nature is believed to permit defect correction for the improvement of crystallization.^24–27^ Furthermore, B–N linkages are also anticipated to incorporate functional groups into COPs, providing exotic application potential for their facile modification.^28,29^

Tetrathiafulvalene (TTF) and TTF derivative (TTFs)-based materials are sulfur-rich compounds, which have been demonstrated to show promising prospects in rechargeable batteries.^30–32^ For example, the Zuo group developed three metal–organic frameworks (MOFs) based on *ortho*-tetrathiafulvalene octabenzoate, which displayed high specific capacity (up to 1249 mA h g^−1^) as lithium-ion battery (LIB) anodes.^33^ Such remarkable achievement has inspired researchers to design various TTF-based products for pursuing high-performance electrodes. Significantly, sodium-ion batteries (SIBs) are of great interest for their low cost and abundant sodium sources. To date, many novel materials such as ether-based electrolytes, metal oxides, and covalent organic frameworks (COFs) have exhibited favorable performance in SIBs.^34–36^ Since TTFs-based electrodes have been barely employed in SIBs, their application in SIBs is highly desirable.

Here, we report the preparation of single crystals of one-dimensional (1D) nanobelts named CityU-26, through the reaction between 4,4′,5,5′-tetrakis(4-(pyridin-4-yl)phenyl)-2,2′-bi(1,3-dithiolylidene) (TTF-py) and 1,4-bis(benzodioxaborole) benzene (BACT). The dative B–N interactions between these building units resulted in the formation of the nanobelts, and hydrogen bonding interactions between the nanobelts gave rise to a three-dimensional (3D) supramolecular structure, as characterized by SCXRD analysis. Given the sulfur-rich central component of TTF-py, we explored the application potential of CityU-26 as anodes in SIBs and found that CityU-26 exhibited a capacity of 365 mA h g^−1^ at 150 mA g^−1^, and the capacity could reach 315 mA h g^−1^ at 750 mA g^−1^. The excellent Na ion storage behaviors of CityU-26 highlight its considerable prospects in electrode materials.

## Results and discussion

**Fig. 1:**
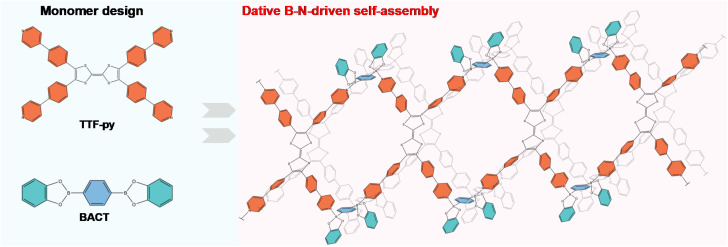
Schematic representation of the design and formation of CityU-26.

TTF-py was synthesized according to the reported procedure with slight modifications (Fig. S1†),^37^ and the linker BACT was prepared through the esterification of boric acid (Fig. S2†). The structures of two building blocks are illustrated in Fig. 1. Briefly, TTF-py provides four pyridine moieties as N-donors, and BACT offers two boron esters as reactive sites. Red single crystals of CityU-26 (Fig. S3†) were obtained by the slow solvent evaporation of a mixture of toluene and methanol at 85 °C overnight. SCXRD measurement indicated that CityU-26 crystallized in the triclinic space group P-1 with lattice parameters of *a* = 16.3894(3) Å, *b* = 17.8532(4) Å, and *c* = 20.0109(5) Å.

**Fig. 2:**
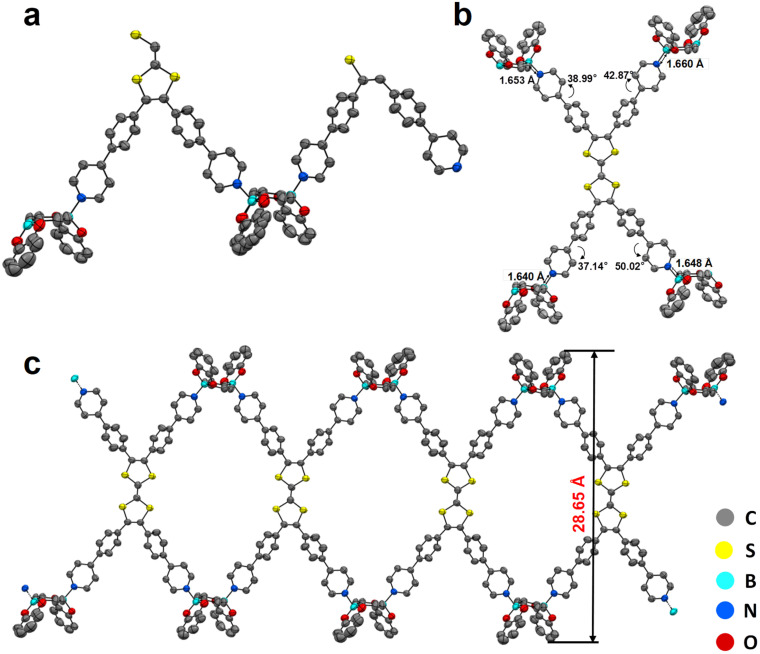
(a) The asymmetric unit of CityU-26. Thermal ellipsoids are drawn at the 50% probability level. Hydrogen atoms and toluene molecules are omitted for clarity. The yellow, red, blue, and cyan balls represent sulfur atoms, oxygen atoms, nitrogen atoms, and boron atoms, respectively. (b) TTF-py monomer and the four BACT units to which it is connected in CityU-26. (c) A single nanobelt with a thickness of 28.65 Å in CityU-26.

**Fig. 3:**
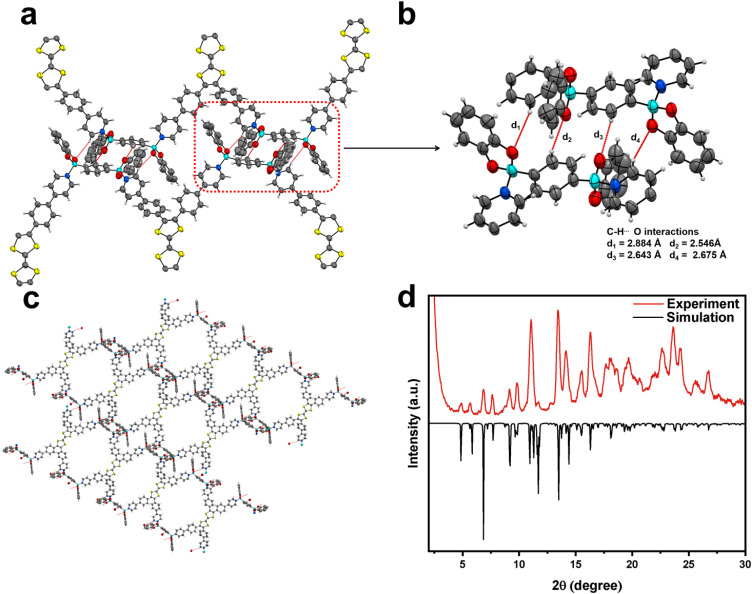
(a) and (b) Illustration of the hydrogen bonding interactions between neighbouring nanobelts. (c) Illustration of the supramolecular structure in CityU-26 constructed by C–H⋯O hydrogen bond interactions, represented by red dashes. (d) Experimental and simulated PXRD patterns of CityU-26.

Within one asymmetric unit of CityU-26 (Fig. 2a), there are two half TTF-py, two BACTs, and two and a half toluene molecules (omitted for clarity). Each TTF-py molecule interacts with four BACT units through B–N bonds, with lengths of 1.640, 1.648, 1.653, and 1.660 Å, in good agreement with those of reported dative B–N bonds (Fig. 2b). As a result of the B–N coordination, four pyridine rings of one TTF-py are twisted with dihedral angles of 38.99°, 42.87°, 37.14°, and 50.02°, respectively. Each BACT is also linked to two pyridines from two TTF-py ligands, thus generating 1D nanobelts (Fig. 2c). One half TTF-py and two half BACT constitute a repetitive unit of the nanobelts, with a measured thickness of 28.65 Å. These nanobelts stack along the *c* axis with a distance of 20.011 Å (Fig. S4†). Besides, carbon–oxygen (C–H⋯O) hydrogen bonding interactions between these nanobelts are observed, with corresponding distances of 2.884, 2.546, 2.643, and 2.675 Å, respectively (Fig. 3a and b). These interactions occur between oxygen atoms (BACTs) and carbon atoms (pyridines and BACTs), thus resulting in the generation of a 3D supramolecular structure and stabilizing CityU-26 (Fig. 3c). In addition, further investigation of the overall structure of CityU-26 indicates a linear arrangement of TTF-Pys connected by flat BACTs, leading to the formation of rhombic channels with dimensions of 6.904 × 13.967 Å^2^ (Fig. S5†).

PXRD analysis was utilized to clarify the purity of the obtained products. The measured PXRD pattern matches the simulated one well, demonstrating the high phase purity of the products (Fig. 3d). Peaks observed at 4.86°, 5.83°, and 6.85° correspond to the (001), (011), and (111) planes, respectively. The nanobelt construction of CityU-26 is attributed to B–N bonding, as confirmed by its Fourier-transform infrared (FTIR) spectrum, where the peak at 1080 cm^−1^ supports the formation of B–N linkages (Fig. S6†). The thermal stability of CityU-26 was determined by the thermogravimetric analysis (TGA) test (Fig. S7†). The initial weight loss of ∼13.8% is ascribed to solvent escape, further confirming the presence of 2.5 toluene molecules per unit of CityU-26. Regardless, CityU-26 exhibits acceptable stability up to 245 °C. We further investigated the UV-vis spectrum of CitU-26 (Fig. S8†). Though BACT exhibits limited absorption, CityU-26 displays evident absorption bands around 930 nm and 1230 nm in the near-infrared region, which may arise from its nanobelt structure.

**Fig. 4:**
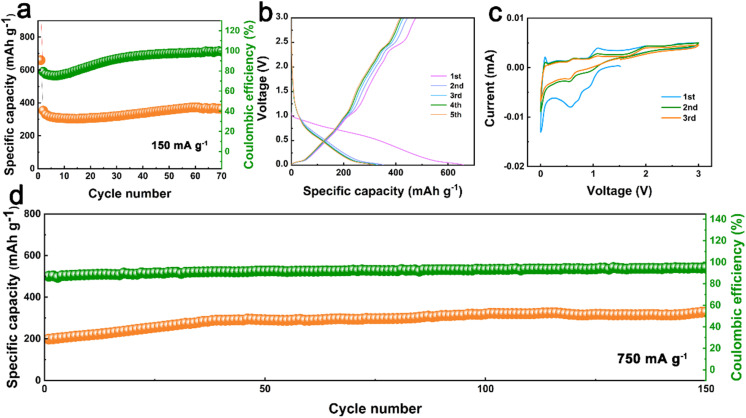
(a) The cyclic performance of CityU-26 at 150 mA g^−1^, (b) charge–discharge voltage profiles of the first 5 cycles under 150 mA g^−1^, (c) CV curves at 0.1 mV s^−1^, (d) cyclic performance at 750 mA g^−1^ of CityU-26 as a SIB anode.

Given the regular arrangement of TTF-pys in CityU-26, where the central unit TTF is sulfur-rich, we explored the potential application of CityU-26 in the electrochemistry field. CR2032 coin cells were assembled to evaluate the energy storage capacity of CityU-26 as SIB anodes, demonstrating the Na ions' excellent de-intercalation ability in rechargeable batteries. The Na ion storage capacity is depicted in Fig. 4. The cycling performance of the anode based on CityU-26 demonstrated cycling stability with a specific capacity of 365 mA h g^−1^ at a current density of 150 mA g^−1^. The low coulombic efficiency in the initial cycles was induced by the generation of a solid electrolyte interphase (SEI) (Fig. 4a). Galvanostatic charge–discharge curves of the first 5 cycles in Fig. 4b displayed a typical discharge platform at around 1.0 and 0.5 V, corresponding to the cathodic peaks of the cyclic voltammetry (CV) curves (Fig. 4c), indicative of the Na ion insertion process. The basically overlapping profiles suggest that the anode can stably accommodate the insertion and extraction of Na ions. The CV curves showed the first 3 cycle scans at a scan rate of 0.1 mV s^−1^ in a voltage range from 0.01 to 3 V. In the first scan curve, the cathodic peaks appear at around 1.0 V and 0.5 V, and a broad peak in the range of 0.5–0.8 V is observed, arising from the formation of an irreversible SEI on the anode surface.^38–40^ The intense cathodic peaks are attributed to the insertion of Na ions into CityU-26. Nevertheless, in the second and third scan curves, observation of cathodic peaks (at 0.5 V and 1.0 V) and anodic peaks (at around 0.6 and 1.1 V) suggests CityU-26 with the two-step insertion and extraction of Na ions. Due to the low electrical conductivity of CityU-26, attributed to the long distances between neighboring nanobelts along the *c* axis (Fig. S4†), the charge and discharge platforms exhibited inconsistencies. This was evident from CV curves near 1 V, where the cathodic peaks located below 1 V, and the anodic peaks were observed above 1 V. Correspondingly, the charging plateau appeared above 1 V, while the discharging plateau was observed below 1 V. This discrepancy resulted in a higher charge capacity contribution at 1 V compared to the discharge capacity. The CV curves of TTF-py are presented in Fig. S9,† where the cathodic peaks located at around 0.5 V and 0.9 V, while anodic peaks appeared at around 0.6 V and 1.1 V. These peaks were consistent with those observed in CityU-26, indicating that the active sites in TTF-py contribute to the efficient storage of Na ions. The CV curves of Super P are also displayed in Fig. S10.† The cathodic peaks and anodic peak mainly appeared at around 0 V, indicating that Super P contributes to the overall capacity at around 0 V of the CityU-26-based anode as well.

After 100 cycles of precycling for activation, the cycling performance of the CityU-26-based anode at the current density of 750 mA g^−1^ is presented in Fig. 4d. The capacity gradually increased in the first 100 cycles because of the activation process. As the sodiation and desodiation process proceeded, the internal active sites were exposed to varying degrees, revealing more metal centers. After 100 cycles, the cycling performance reached a stable state, with the specific capacity maintained at approximately 315 mA h g^−1^ and the coulombic efficiency approaching 100%. Since CityU-26 is insufficiently conductive as SIB anodes, dynamic transmission performance would deteriorate when large currents are applied. Therefore, increased energy loss during the charging process produced coulombic efficiency lower than 100%. In addition, the GITT curves and the corresponding Na^+^ diffusion coefficients were tested and are shown in Fig. S11.† The changes in the anode impedance and the fitting equivalent circuit model of electrochemical impedance spectroscopy (EIS) are depicted in Fig. S12.† During the activation processes, the capacity of the anode increased, accompanied by a decrease in impedance. The fitting of the EIS plots revealed a slight decrease in anode impedance from 530 Ω to 460 Ω, indicating an improvement in charge transfer by the activation. We also studied the cycling performance of anodes based on Super P and TTF-py at 150 mA g^−1^, respectively (Fig. S13 and S14†). Both anodes exhibited relatively low specific capacities. Given that the capacity of super P is approximately 100 mA h g^−1^ as anode materials for SIBs, the capacity contribution from Super P in the CityU-26-based anode should be around 50 mA h g^−1^ (=100 mA h g^−1^ × (30 wt% super P/60 wt% CityU-26)). Therefore, the corresponding capacity contribution from CityU-26 was about 315 mA h g^−1^ at 150 mA g^−1^, much higher than that of super P, indicating that CityU-26 played a vital role in sodium storage. The theoretical specific capacity for sodium storage in CityU-26 is 296.8 mA h g^−1^ (*C* = *n* × (*F* × 1000)/(*M* × 3600), *F* = 96 485.3321233100184 C mol^−1^, *M* = 1444.82), in good agreement with the measured values since the layered structures of CityU-26 could facilitate the intercalation of Na^+^ ions to enhance its storage capacity. Thus, the impressive sodium storage properties of the CityU-26-based anode are attributed to the nanobelt construction and the incorporation of TTF-py, which provides sulfur atoms as active sites for Na ion storage.^41,42^ The high electron cloud density of sulfur atoms should give rise to the accumulation of Na ions around sulfur atoms in the nanobelts, and the rhombic channels of CityU-26 provide enough space for sodiation and desodiation, thus endowing CityU-26 with a high specific capacity as SIB anodes. The excellent electrochemical performance of CityU-26 demonstrates its outstanding Na ion storage behavior. The successful functionalization of the B–N nanobelts inspires us to introduce diverse functional groups and enrich the B–N polymer family, paving a promising way to develop efficient energy materials.

## Conclusions

In summary, we have developed a 1D single-crystal nanobelt CityU-26 through the slow evaporation of the solution mixture containing TTF-py and BACT at high temperatures. The B–N coordination between TTF-py and BACT produced nanobelts with rhombic channels, while hydrogen bonding interactions between these nanobelts led to the formation of a 3D supramolecular structure, as confirmed by SCXRD. The incorporation of the functional group TTF endowed CityU-26 with excellent Na ion storage capability as SIB anodes. CityU-26 exhibited a high specific capacity of 365 mA h g^−1^ at 150 mA g^−1^ and demonstrated stable cycling performance at a high current density of 750 mA g^−1^. The outstanding electrochemical performance of CityU-26 demonstrates the feasibility of the functionalization of B–N polymers, providing some guidelines for designing efficient storage materials.

## Data availability

All experimental procedures and characterisation data can be found in the article or in the ESI.† CCDC 2362109 contains the supplementary crystallographic data for this paper. These data can be obtained free of charge from The Cambridge Crystallographic Data Centre *via*http://www.ccdc.cam.ac.uk/data_request/cif.

## Author contributions

Y. Ren, C. S. Lee, and Q. Zhang designed the research. X. Wang and Y. Zhang conducted the experiments. L. Zhang analyzed the crystallographic data. Q. Gu conducted manuscript revision.

## Conflicts of interest

There are no conflicts to declare.

## Supplementary Material

SC-015-D4SC06300G-s001

SC-015-D4SC06300G-s002
