# Supplementary material for: A tetrathiafulvalene-containing covalent organic nanobelt: preparation, crystal structure and application for sodium-ion batteries
Source: Chem Sci. 2024 Nov 14;15(48):20397–402. doi: 10.1039/d4sc06300g (PMC11580028; doi:10.1039/d4sc06300g)
Supplement: SC-015-D4SC06300G-s001 [file SC-015-D4SC06300G-s001.pdf]

Supplementary Information (SI) for Chemical Science.
This journal is © The Royal Society of Chemistry 2024

Supporting Information

A Tetrathiafulvalene-Containing Covalent Organic Nanobelt: Preparation, Crystal Structure and Application for Sodium-Ion Battery

Xin Wang, Yuchan Zhang, Lei Zhang, Qianfeng Gu, Qi Liu, Yang Ren, Chun Sin Lee, and Qichun Zhang*

1. Materials and methods

All chemicals and solvents were used directly without purification if not specified. PXRD pattern was collected by Rigaku X-ray Diffractometer SmatlabTM 9kW. Thermal analysis was conducted on PerkinElmer Simultaneous Thermal Analyzer (STA) 6000 from 30 to 800 °C at a heating rate of 10 °C min^{-1} under N_2. UV-Vis spectra from 300 to 1500 nm were carried out on Hitachi UH4150 UV-VIS-NIR Spectrophotometer. FTIR spectra (4000 to 500 cm^{-1}) were tested on a Perkin Elmer Spectrum II.

SCXRD test of **CityU-26** was conducted on the Rigaku X-ray Single Crystal Diffractometer System (Rigaku SmartLab 9kW-Advance) at room temperature using the monochromatized wavelength (Cu Kα) = 1.54184 Å. The crystal structures were solved and refined by full matrix least-squares methods against F2 using the SHELXL-2013-2 program package and Olex-2 software. All non-hydrogen atoms were refined with anisotropic displacement parameters and hydrogen positions were fixed at calculated positions and refined.

Synthesis of CityU-26

5 mg TTF-py and 3mg BACT were added into the mixture of 3 mL toluene and 1 mL methanol, followed by sonication to get a clear solution. Red crystals could be obtained after the slow evaporation of the solution at 85 °C overnight.

Electrochemical measurements

CityU-26, super P and PVDF binder were mixed with the weight ratio of 6:3:1. Then the slurry after stirring 12 h was evenly coated on the Al foil and dried at 80 °C in vacuum oven overnight to obtain the pole piece.

The CR2032 coin cells were assembled at the glovebox to investigate the electrochemical performance of **CityU-26** in SIBs. Na metal as the counter electrode, glass fiber as the separator and 1M NaPF6 in DIGLYME as the electrolyte. The galvanostatic cycling performance measurement was executed on the Neware battery test system CT-4008. The CV test was

carried out with the voltage range between 0.01 and 3 V at the scan rate of 0.1 mV s^{-1}. All electrochemical measurements were carried out at 25 °C.

2. Synthesis of Monomers

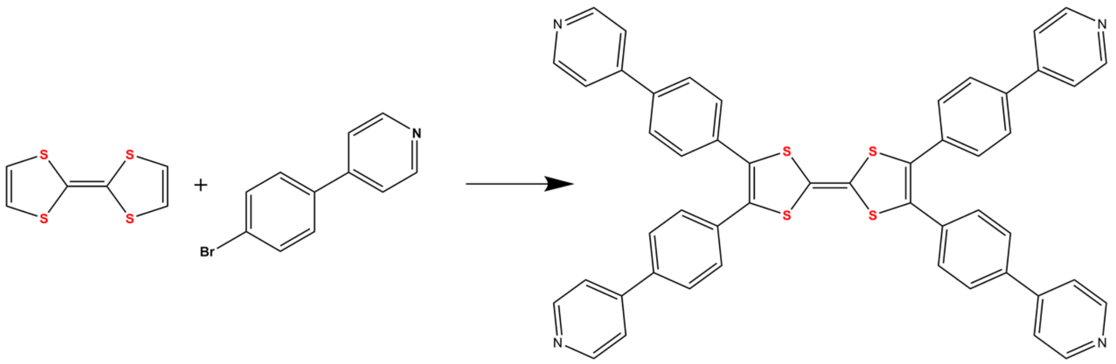

Fig. S1. Synthetic route to TTF-py.

To 20 mL distilled dioxane was added palladium acetate (82 mg), tri-tert-butylphosphonium tetrafluoroborate (320 mg), and cesium carbonate (2.40 g). Then, the suspension was heated at 90 °C for 10 min under argon, followed by the addtion of an argon-degassed dioxane including tetrathiafulvalene (300 mg, 1.46 mmol) and 4-(4-Bromo-phenyl)-pyridine (1.7 g). The reaction was stirred under reflux for 72 h. After cooling, the products were extracted from chloroform and purified by chromatography on silicon gel.

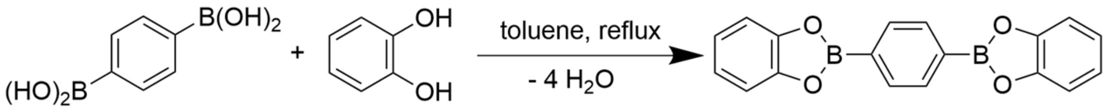

Fig. S2. Synthetic route to BACT.

Benzene-1,4-diboronic acid (0.5055 g) and catechol (0.72 g) were added to a 100-mL flask with 40 mL toluene. The mixture was heated under reflux overnight with a Dean−Stark trap filled half full with 3 Å sieves. White needle-like crystals were obtained by solvent evaporation under reduced pressure, followed by sublimation to remove excess catechol.

3. Characterization

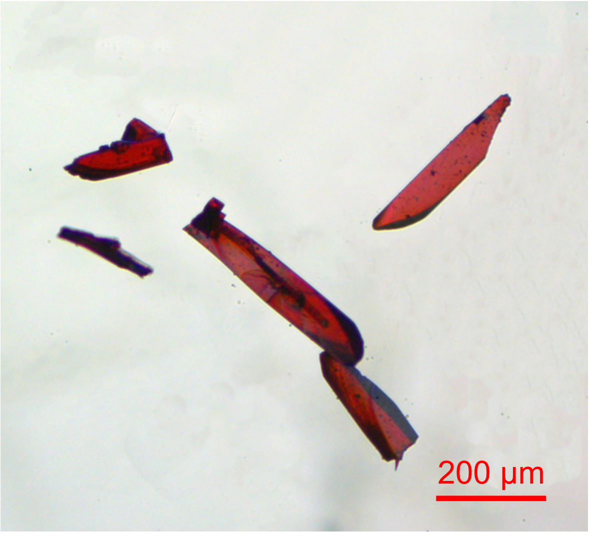

Fig. S3. Optical image of **CityU-26** crystals.

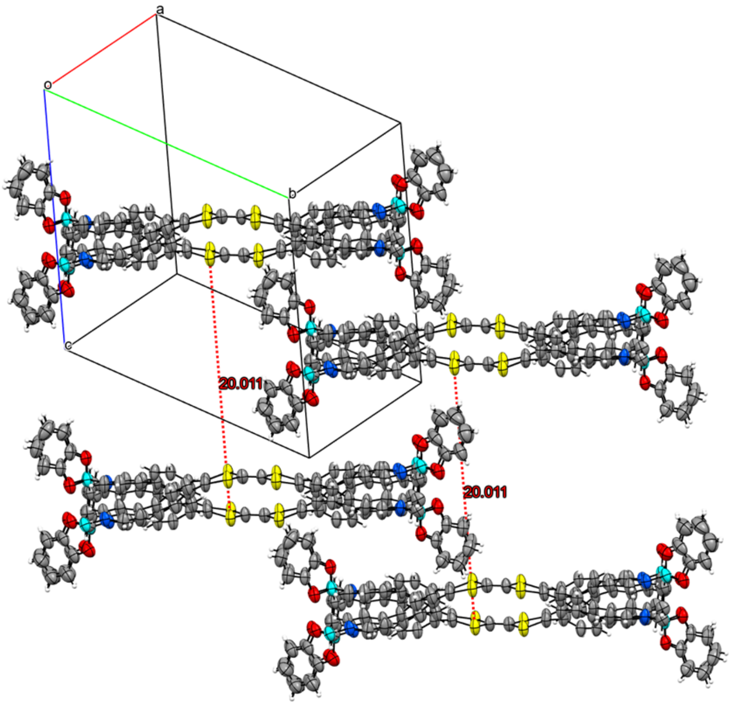

Fig. S4. The packing diagram of the nanobelts in **CityU-26**.

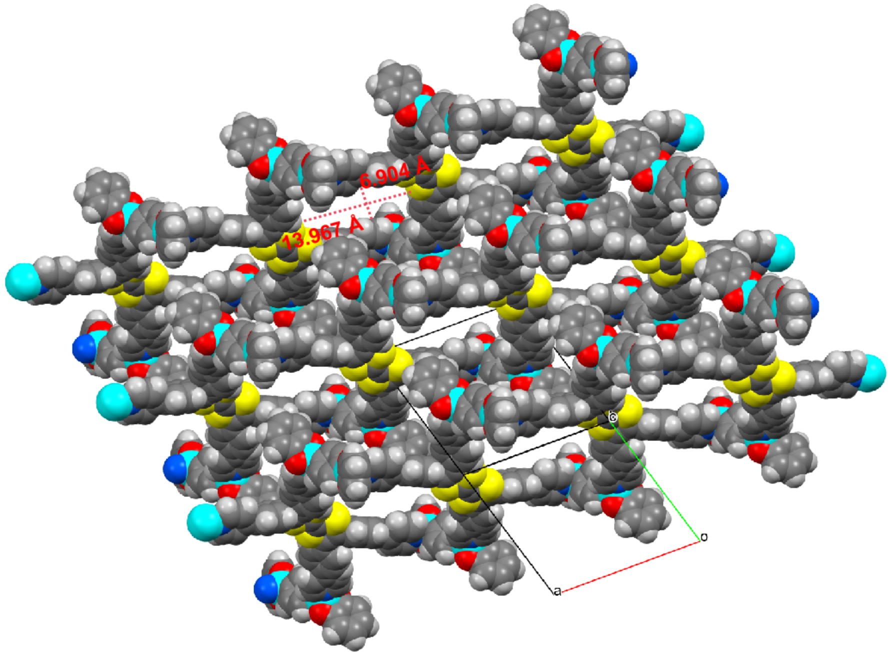

Fig. S5. Rhombic channels in **CityU-26**.

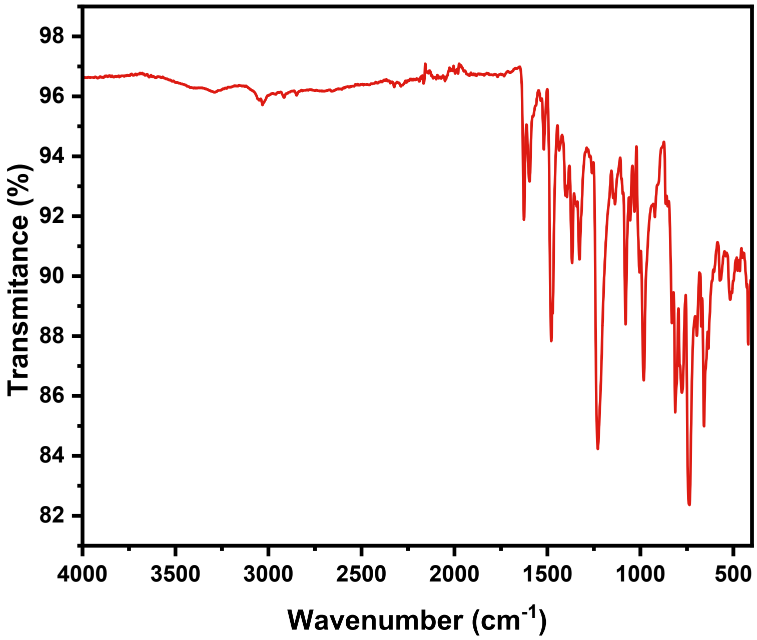

Fig. S6. FTIR spectrum of **CityU-26**.

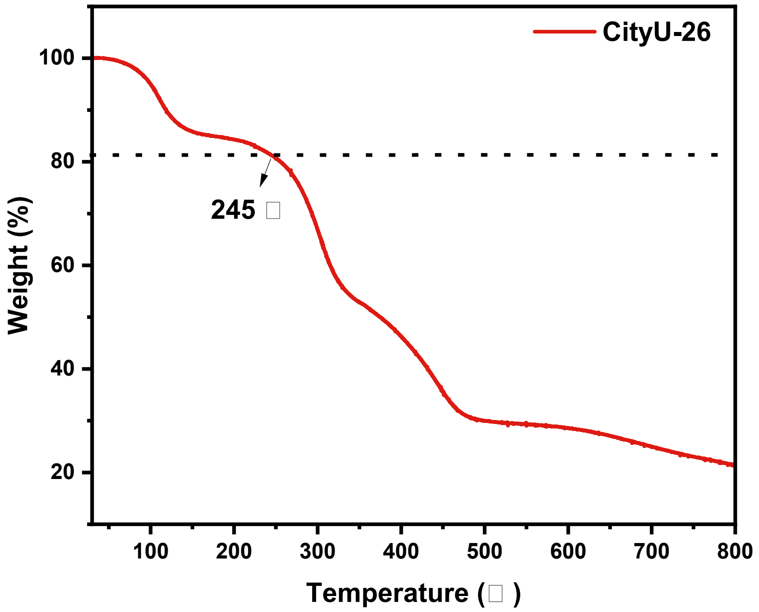

Fig. S7. TGA curve of **CityU-26**.

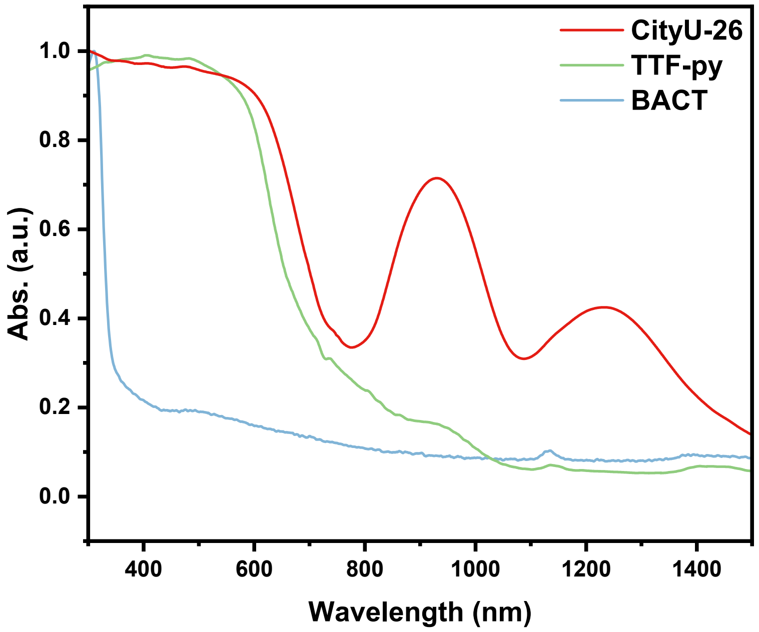

Fig. S8. UV-Vis spectra of **CityU-26**, TTF-py and BACT.

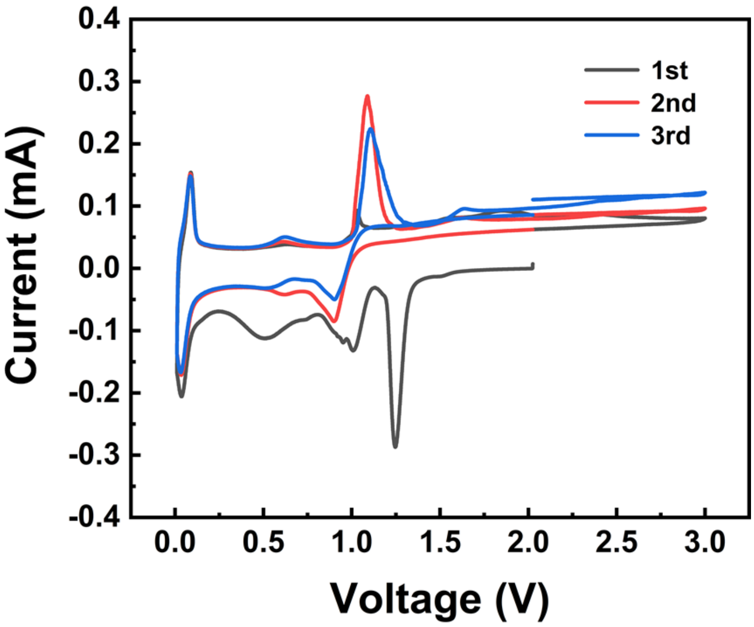

Fig. S9. The CV curves of TTF-py.

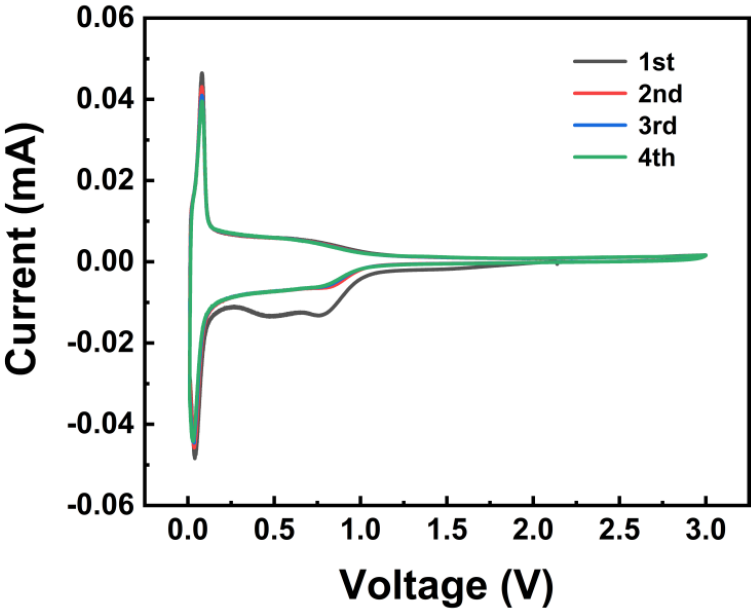

Fig. S10. The CV curves of Super P.

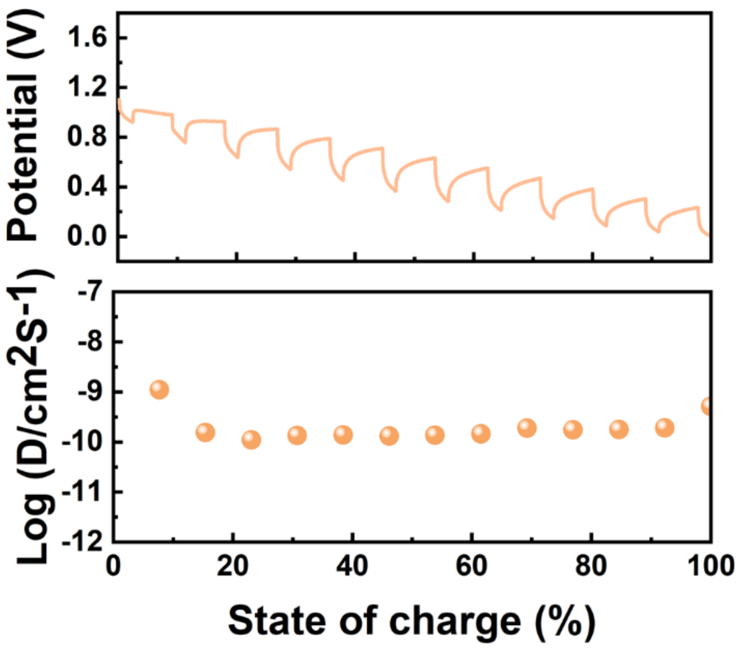

Fig. S11. The GITT curves and the corresponding Na^+ diffusion coefficients.

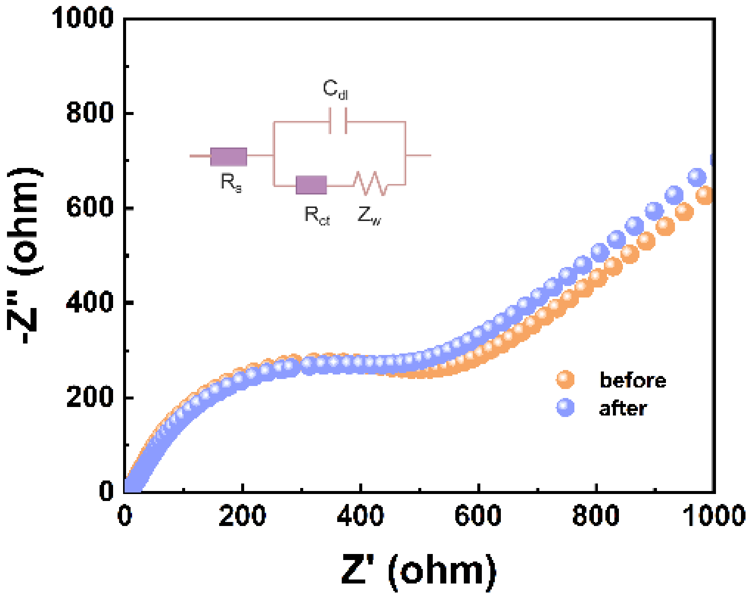

Fig. S12. Impedance of the **CityU-26**-based anode before and after cycling.

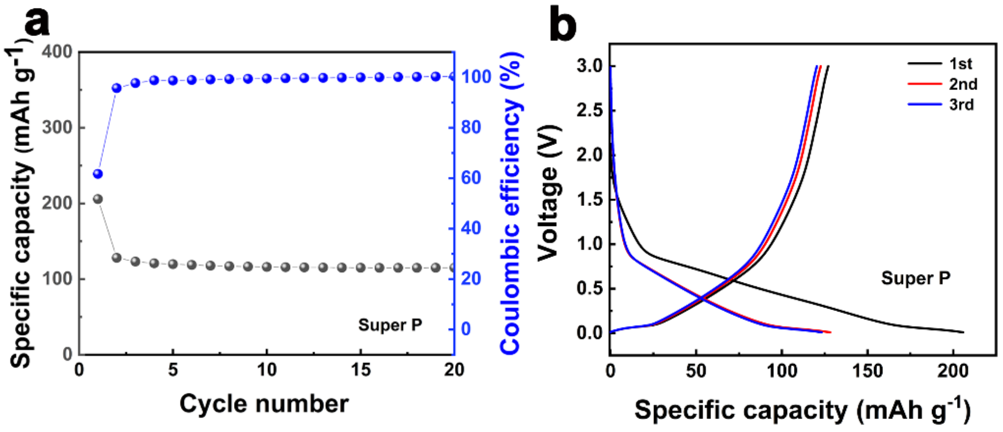

Fig. S13. The cyclic performance of Super P at 150 mA g^{-1}, (b) Charge-discharge voltage profiles of carbon black Super P.

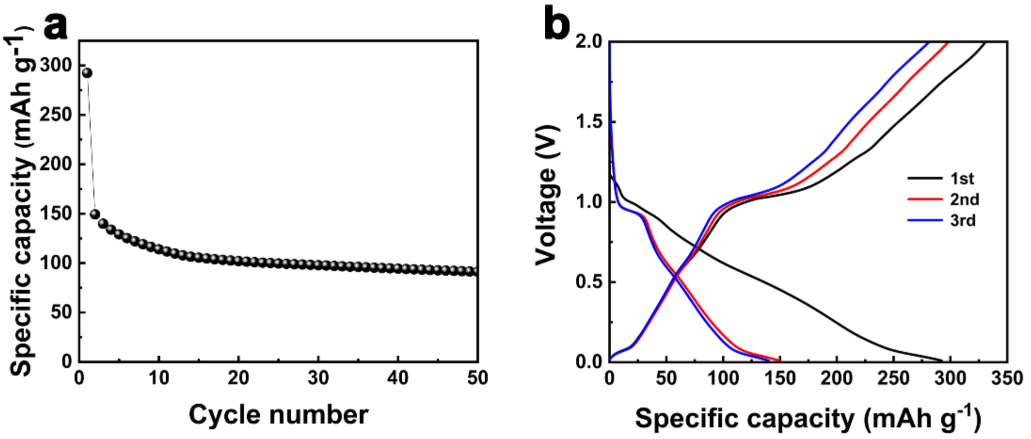

Fig. S14. The cyclic performance of TTF-py at 150 mA g^{-1}, (b) Charge-discharge voltage profiles of TTF-py.

Table S1. Crystal data and structure refinement of **CityU-26**.

	**CityU-26**
**Empirical formula**	$C_{86}H_{56}B_4N_4O_8S_4$
**Formula weight**	1444.82
**Crystal system**	triclinic
**Space group**	*P*-1
a **(Å)**	16.3894(3)
b **(Å)**	17.8532(4)
c **(Å)**	20.0109(5)
α **(°)**	65.460(2)
β **(°)**	79.648(2)
γ **(°)**	73.025(2)
V **(Å^3)**	5082.8(2)
Z	2
D_{calc}**(g • cm^{-3})**	0.944
**Abs.coeff.(mm^{-1})**	1.217
F**(000)**	1496
**Reflns collected**	55553
**GOFon** F^2	1.067
**Final R indexes [I>=2σ (I)]**	R1 = 0.1041, wR_2 = 0.3175
**Final R indexes [all data]**	R1 = 0.1304, wR_2 = 0.3509

Table S2. Selected bond distance (Å) of **CityU-26.**

Atom1	Atom2	Length/Å
O1	B1	1.494(5)
O2	B1	1.446(6)
N2	B1	1.640(5)
C31	B1	1.594(6)
O6	B2	1.41(2)
O5	B2	1.453(17)
N3	B2	1.653(5)
C36	B2	1.585(6)
O8	B3	1.501(5)
O7	B3	1.458(6)
N4	B3	1.660(5)
C82	B3	1.574(6)
O10	B4	1.470(6)
O9	B4	1.475(6)
C85	B4	1.596(6)
N1	B4[2]	1.648(5)
B4	N1[3]	1.648(5)
B2	O3	1.539(16)
B2	O4	1.525(19)
C62	S1[1]	1.751(4)
C12	S2[1]	1.761(4)

[1]1-X,-Y,2-Z;[2]2+X,-1+Y,1+Z;[3]-2+X,1+Y,-1+Z

Table S3. Selected bond angles (°) of **CityU-26.**

Atom1	Atom2	Atom3	Angle/°
C23	N2	B1	121.4(3)
C24	N2	B1	120.2(3)
C25	O2	B1	105.8(3)
C26	O1	B1	104.8(3)
C43	O6	B2	107.8(11)
C44	O5	B2	104.7(10)
C49	N3	B2	120.9(3)
C50	N3	B2	121.1(3)
C73	N4	B3	119.4(3)
C74	N4	B3	122.5(3)
C75	O7	B3	106.5(3)
C76	O8	B3	105.2(3)
C87	O9	B4	104.2(4)
C88	O10	B4	106.9(4)
C2	N1	B4[2]	120.6(3)
C3	N1	B4[2]	121.2(3)
C37	O4	B2	105.3(9)
C38	O3	B2	104.8(11)
O10	B4	N1[3]	107.2(3)
O9	B4	N1[3]	105.9(4)
C85	B4	N1[3]	107.4(3)
O1	B1	N2	105.3(3)
O2	B1	N2	105.9(3)
C31	B1	N2	111.1(3)
O6	B2	N3	107.6(9)
O5	B2	N3	101.2(8)
C36	B2	N3	108.9(3)
O4	B2	N3	104.1(9)
O3	B2	N3	107.6(8)
O8	B3	N4	104.3(3)
O7	B3	N4	106.5(3)
C82	B3	N4	109.1(3)

[1]1-X,-Y,2-Z;[2]2+X,-1+Y,1+Z;[3]-2+X,1+Y,-1+Z
